# Supplementary material for: SyNDI: synchronous network data integration framework
Source: BMC Bioinformatics. 2018 Nov 6;19:403. doi: 10.1186/s12859-018-2426-5 (PMC6219086; doi:10.1186/s12859-018-2426-5)
Supplement: Supplementary file 5 — Files of the example presented in Additional file 4. This additional file is a zip package that contains all files used in Additional file 4. Detailed descriptions of each of these files are presented in a README file included in this zip package. (GZ 30411 kb) [file 12859_2018_2426_MOESM5_ESM.gz › SyNDITools/Meme2Fimo/meme_4.9.0_4.tar.gz!/meme_4.9.0/website/html/help_alphabet.html]

MEME Suite - Alphabet


|  |  |
| --- | --- |
|  | MEME Suite The MEME Suite web application requires the use of JavaScript   Javascript doesn't seem to be available on your browser. DNA or protein sequences are accepted. The sequences must all be of the **same type**, either protein or DNA.  - **Protein sequences** should use the standard IUPAC   alphabet: **ACDEFGHIKLMNPQRSTVWY**.       They may also contain the ambiguous letters **"BUXZ"**, which   are converted to **"X"** and treated as "unknown". - **DNA sequences** should use the standard DNA alphabet:   **ACGT**.       They may also contain the ambiguous letters **"BDHKMNRSUVWY"**,   which will be converted to **"X"** and treated as   "unknown".  **Note:** If none of the sequences in your dataset contain any of the letters **"EFILPQXZ"**, it will be assumed that your sequences are DNA. You can force them to be interpreted as protein sequences by adding an **"X"** to the end (or beginning) of one of the sequences in your dataset. |
